# A Porcine Congenital Single-Sided Deafness Model, Its Population Statistics and Degenerative Changes

**DOI:** 10.3389/fcell.2021.672216

**Published:** 2021-06-11

**Authors:** Wei Ren, Cong Xu, Fan-jun Zheng, Ting-ting Lin, Peng Jin, Yue Zhang, Wei-wei Guo, Chuan-hong Liu, Xiao-yang Zhou, Lu-lu Wang, Yong Wang, Hui Zhao, Shi-ming Yang

**Affiliations:** ^1^College of Otolaryngology Head and Neck Surgery, Chinese PLA General Hospital, Beijing, China; ^2^National Clinical Research Center for Otolaryngologic Diseases, Beijing, China; ^3^Key Lab of Hearing Science, Ministry of Education, Beijing, China; ^4^Beijing Key Lab of Hearing Impairment for Prevention and Treatment, Beijing, China; ^5^Department of Laboratory Animal Science, College of Basic Medical Science, Army Medical University, Chongqing, China; ^6^Department of Human Genetics, Emory University School of Medicine, Atlanta, GA, United States

**Keywords:** porcine (pig) model, deafness (hearing loss), ABR, DPOAE, pathology

## Abstract

**Objective:**

To describe and study the population statistics, hearing phenotype, and pathological changes of a porcine congenital single-sided deafness (CSSD) pedigree.

**Methods:**

Click auditory brainstem response (ABR), full-frequency ABR, and distortion product otoacoustic emission (DPOAE) were used to assess the hearing phenotype of the strain. Tympanogram was used to assess the middle ear function since birth. Celloidin embedding–hematoxylin–eosin (CE-HE) stain and scanning electron microscopy (SEM) were used to study the pathological changes of cochlear microstructures. Chi-square analysis was used to analyze the relation between hearing loss and other phenotypes.

**Results:**

The mating mood of CSSD with CSSD was most efficient in breeding-targeted CSSD phenotype (47.62%), and the prevalence of CSSD reached 46.67% till the fifth generation, where 42.22% were bilateral hearing loss (BHL) and 9.00% were normal hearing (NH) individuals. Hearing loss was proved to have no relation with coat color (*P* = 0.0841 > 0.05) and gender (*P* = 0.4621 > 0.05) by chi-square analysis. The deaf side of CSSD offspring in the fifth generation had no relation with that of their maternal parent (*P* = 0.2387 > 0.05). All individuals in this strain exhibited congenital severe to profound sensorineural hearing loss with no malformation and dysfunction of the middle ear. The good hearing ear of CSSD stayed stable over age. The deaf side of CSSD and BHL presented cochlear and saccular degeneration, and the hair cell exhibited malformation since birth and degenerated from the apex to base turn through time. The pathology in BHL cochlea progressed more rapidly than CSSD and till P30, the hair cell had been totally gone. The stria vascularis (SV) was normal since birth and degenerated through time and finally exhibited disorganization of three layers of cells.

**Conclusion:**

This inbred porcine strain exhibited high and stable prevalence of CSSD, which highly resembled human non-syndromic CSSD disease. This porcine model could be used to further explore the etiology of CSSD and serve as an ideal tool for the studies of the effects of single-sided hearing deprivation on neural, cognitive, and behavioral developments and the benefits brought by CI in CSSD individuals.

## Introduction

Single-sided deafness (SSD) is defined as profound sensorineural hearing loss in one ear with normal hearing on the opposite side. Congenital SSD (CSSD) often refers to those who did not pass the newborn hearing screening and then was diagnosed with unilateral hearing loss at birth. The incidence of sensorineural hearing loss is estimated to be 1.86 per 1,000 newborns; among them, 30–40% are unilateral (Fitzpatrick et al., 2017; van Wieringen et al., 2019), but the CSSD incidence varies among researches because populations of different ages were involved, for children from 6 to 19 years old. The estimated CSSD incidence was 0.7–0.8% (Ross et al., 2010). In South Korea, the prevalence of unilateral hearing loss (UHL) was 9.31%, among which SSD accounts for 5.98% in the population over 12 years old; however, the prevalence of CSSD was unknown (Jun et al., 2015). Because of the lack of international or regional epidemiology study, the incidence of CSSD needs further study.

Many researches had tried to find etiologies of CSSD; some identified risk factors including cochlear nerve deficiency (Clemmens et al., 2013; Lipschitz et al., 2020), congenital cytomegalovirus, congenital inner ear malformation, and bacterial and viral meningitis, but more than 60% of the CSSD was of unknown etiology (van Wieringen et al., 2019). Unilateral and asymmetric hearing loss in a Waardenburg Syndrome Type 2 (WS2) pedigree was reported to be caused by mutation in *KIT* or *KITLG*. KIT-KITLG signaling pathway and MITF were suggested to mutually interact in the migration process of melanocyte from the neural crest to stria vascularis (SV). The imbalanced migration and distribution of melanocytes in stria vascularis might be cause by laterality of hearing (Zazo Seco et al., 2015; Hamadah et al., 2019). However, most cases of CSSD in the clinic were non-syndromic and reported to be not correlated with other systematic symptoms.

Besides the high prevalence, CSSD gradually catches clinical physicians’ attention because there is a growing consensus that children with CSSD have difficulties in hearing, sound localization, and speech discrimination in a noisy environment. Moreover, CSSD also negatively influences the neural, cognitive, language, and behavioral development and neural network working mode (Kral and O’Donoghue, 2010; Maslin et al., 2013). A large body of researches had verified that the duration and onset of UHL were two key factors impacting the auditory restoration, cochlear implantation (CI) outcome, and cortical speech processing (Kral and Sharma, 2012; Vanderauwera et al., 2020). Clinically, CI might be the only way to restore hearing, but its outcome was controversial. A longitudinal study on six CSSD infants with early CI intervention demonstrated that children showed beneficial outcome in language, cognitive development, and hearing compared to non-implanted samples (Sangen et al., 2019). Additionally, early CI also helped avoid neurofunctional dominance of the hearing ear and would be beneficial to the neural development in deaf cats (Kral et al., 2013), indicating early CI in the case of CSSD.

Few clinical studies separate CSSD and acquired SSD (ASSD) in neuroscientific studies. The mechanism and the onset of neural reorganization might differ for CSSD and ASSD, which should be considered (Vanderauwera et al., 2020). Since clinical research cannot reveal the pathological changes and the observation period for brain and auditory system function changes is usually very long, researchers referred to animal models. Different species of animals have been used in the hearing research, including chinchilla, white deaf cats, mouse, rat, and dogs with pigmentation. Various methods have been tried to establish the SSD animal model, among which cochlear ablation is the most often used method. Other ways include local injection of high dose of gentamicin or neomycin at early postnatal days to cochleae to mimic the congenital SSD to study the impact of monaural hearing deprivation on cortical development or deafening adult animals by injecting drugs to the middle ear, posterior canal, and round window at different ages to study the impact of sudden SSD on the trajectory changes of cortical, visual, and language processing (Jakob et al., 2016; Liu et al., 2016; Banakis Hartl et al., 2019; Cheng et al., 2019; Ding et al., 2020; Zhong et al., 2020).

The postnatal artificial SSD could not fully mimic congenital SSD, and the influence of CSSD on cortical development started long before birth. How imbalanced sound signal input affect the neural development since embryo stage remains unknown because of lack of CSSD animal models. Congenitally unilateral deaf animal was reported in feline, canine, and horse breeds. Various reports indicated that deaf white cats (DWCs) were feline homolog of the human Waardenburg syndrome (Schwartz and Higa, 2009) because the coat and iris pigmentation were correlated with hearing loss and several relating genes had been reported, like *PAX3* and *KIT* (David et al., 2014). Andrej Kral explored how unilateral hearing affected cortex plastic reorganizations by using two CSSD white cats (Kral et al., 2013), which were reported and inbred by Heid et al. (1998). However, the CSSD phenotype in cats were very rare and appeared occasionally instead of in a stable inheritable mood. Congenital canine deafness had been observed since 1896 in over 80 breeds (Strain, 2004) with prevalence from 7.0 to 32.3%, among which CSSD accounted for 1.3–18.0% (Rak and Distl, 2005). Most studies focused on the Dalmatian because of its highest hearing loss prevalence with approximately 5.3–8% bilateral deafness and 9.4–21.9% CSSD, or total of up to 30% affected (Strain et al., 1992; Rak and Distl, 2005). Hearing loss in most not all canine breeds also positively corelated with blue iris and coat color pigmentation (Famula et al., 1996). However, the inheritance mechanism and responsible genes remained unknown; the most possible candidate genes include merle (M locus) and *piebald* allele (S locus), which would influence the differentiation and migration of melanocytes in cochlea during embryogenesis (Rak and Distl, 2005). People also failed to inbreed a canine pedigree with stable prevalence of deafness.

Although the above animal breeds exhibited high prevalence of deafness, however, few hereditary components were verified, and nearly all breeds mentioned above mimicked the phenotypes of human Waardenburg syndrome, and the prevalence of CSSD was casual. In our study, we describe a naturally occurring inbred CSSD porcine pedigree with high and stable prevalence of CSSD. Auditory physiology and pathological presentations of different hearing phenotypes in the pedigree were uniform and detailly described.

## Materials and Methods

### Animals

All animals of this inbred Bama Miniature pig pedigree in this paper were provided by the Laboratory Animal Science Center of College of Basic Medicine in the Army Military Medical University (Chongqing, China). All animals were raised in a standard pathogen-free (SPF) condition. Animals younger than P30 were raised in the Lab Animal Science Center in Chongqing; animals older than P30 were delivered to the Lab Animal Center of PLA General Hospital and being raised in the same condition.

### Anesthesia

In all experiments, animals were anesthetized with 1.5–5% Isoflurane in 3:3 mixture of oxygen and air by inhalation machine for animal use (Medical Supplies and Services Int. Ltd., United Kingdom). Animals were put on a heating pad to maintain body temperature.

### Click-ABR Tests

Since the full-frequency auditory brainstem response (ABR) measurement would take about 2 h, pigs under P30 could not stand long-time anesthesia. Therefore, for the pigs younger than P30, only stimulus click (Intelligent Smart EP, United States) was used to diagnose if they are deaf or not, and hearing tests were taken in a small sound-proof booth for animals. Insert ear plug was put into the ear and sealed the external ear canal. Ground electrode was put at the apex nasi; reference electrodes were put in the ipsilateral earlobes of the tested ear, and recording electrode was inserted into the skin of the calvaria along the centerline. The click ABR tests only took about 10 min each pig.

### Full-Frequency ABR Tests

Tucker Davis Technology RZ6 (TDT RZ6) was used to apply the full-frequency—from 1 to 32 kHz—ABR tests in pigs over P30. Electrodes were put in the same position as described above. This part of hearing tests was conducted in a standard soundproof booth. A loudspeaker (MF1 2356) was put in the external ear canal meatus, and the untested ear was masked by a calibrated 60 dB SPL white noise by an inserted earphone.

### Tympanogram and DPOAE

Titan (Interacoustics, Denmark) was used to do the tympanometry, which reflected the function of the middle ear, and parameters like middle ear compliance and volume were obtained. Distortion product otoacoustic emission (DPOAE) from 500 to 10 kHz was measured by Titan, which reflected the function of outer hair cells (OHCs). Tympanogram and DPOAE were tested at P1 and P30.

### Celloidin Embedding–Hematoxylin–Eosin Stain and Scanning Electron Microscopy

Celloidin embedding–hematoxylin–eosin (CE-HE) stained cochlear section and scanning electron microscopy (SEM) samples were made following the methods described in our previous studies. Animals were sacrificed in accordance with the Care and Use of the of Laboratory Animals. Cochleae were extracted from the temporal bone within 10 min and being postfixed in 4% paraformaldehyde (CE-HE) or 2.5% glutaraldehyde (SEM) at 4°C overnight. After being washed in 1% phosphate-buffered saline (PBS) three times, each time 10 min, the cochleae were shifted into 10% ethylenediaminetetraacetic acid (EDTA) solution for decalcification at room temperature (RT) on a shaker for 2 weeks.

For the HE staining, the cochleae were dehydrated using graded ethanol (50, 75, 80, 90, 95, and 100%), and each grade would take 2 days. Then, the cochleae were transferred into graded celloidin (2.5, 5.0, 8, 10, 12.5, and 15%), and each grade would take 7 days at RT. The cochleae were embedded in 15% celloidin in a glass dish for 1–2 months until solidification. Then, the samples were put into 75% ethanol for 2–5 days. The samples were sectioned (15 μm each slice) using a freezing microtome (Leica CM1900) and stained with hematoxylin and eosin. The images were visualized and captured using a Leica DMI3000 microscope. For the SEM, decalcified cochleae were postfixed for 2 h in 1% osmium at RT, dehydrated in graded ethanol (50, 75, 80, 90, 95, and 100%), treated with 2% tannin acid for 2 h at RT, rinsed in 0.24 M phosphate buffer (pH 7.4) for 2 h at RT, and dried in a critical point dryer (HCP-2, Hitachi) using liquid CO_2_. Fixed sections were then coated using a sputter coater and examined under a scanning electron microscope (Helios Nanolab 600i).

### Spiral Ganglion Cell Counting of the CE-HE Specimen

Spiral ganglion cells were counted, using the CE-HE specimen under a Leica DMI3000 light microscope. Two cochleae of each hearing phenotype were counted; 12 serial sections, 15 μm each specimen, containing modiolus were counted, and for each specimen, apical, middle, and basal turn ganglion cells were counted, respectively. The mean values of the above 12 serial sections at each turn were used as the ganglion cell counting number for each turn. Only the cells with clear nucleus were counted (Supplementary Figure 4).

### Statistical Methods

Prism GraphPad 8.4.0 was used to do statistical analysis and draw graphs. Chi-squared test was used to analyze the possible hereditary mode. Unpaired *t*-test was used to analyze the statistical difference of tympanogram parameters and ganglion cell counting number of each group compared to the normal group, and *P* < 0.05 was considered as statistical different.

### Ethical Approval

All experiments and procedures in this paper were conducted under the guidelines of the Care and Use of the Laboratory Animals and approved by both the Animal Ethics Committee of Army Military Medical University and PLA General Hospital.

## Results

### The Mating Strategy of CSSDs Female With Male Individuals Was the Most Efficient Way to Stabilize the Pedigree

**FIGURE 1:**
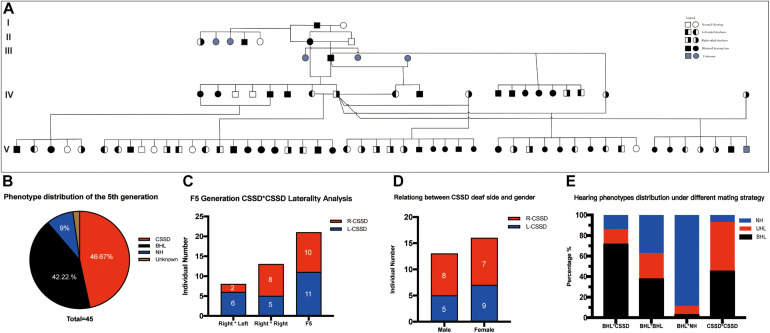
Congenital single-sided deafness (CSSD) pedigree information. **(A)** CSSD pedigree information that consists five generations. **(B)** Hearing phenotype distribution of the fifth generation with different mating strategies. **(C)** Relation of the deaf side between CSSD siblings and their parents. **(D)** Relation between CSSD deaf side and gender. **(E)** Hearing phenotypes distribution under a different mating strategy. L-CSSD, left-sided CSSD; R-CSSD, right-sided CSSD. The individuals with red star in Panel **(A)** were selected to do whole genome resequencing.

Figure 1A showed the family tree of this porcine pedigree. In order to explore the most efficient mating strategy to build up the pedigree and rise the occurrence rate of targeted hearing phenotypes, we tried different mating strategies to explore the phenotype distribution. Under the strategy of CSSD mating with CSSD individuals, the number of targeted CSSD phenotype reached the highest with 20 CSSDs accounting for 47.62% of all offspring, 19 bilateral hearing losses (BHLs) for 45.24%, and only 3 NHs for 7.14%. When CSSD was mating with BHL, the BHLs (10/71.42%) exceeded CSSDs (2/14.28%) by four times. In the mode of BHL mating with BHL, BHL equaled NHs by six (37.5%), and four CSSDs accounted for about 25% of the offspring. When BHL mated with NH, 31 (88.57%) were NHs with only 3 (8.57%) CSSDs and 1 (2.86%) BHL (Figure 1E and Table 1). Thereafter, we mated CSSDs in the fourth generation to get the fifth generation for further studies.

**TABLE 1:** The Iris color and the hearing loss in 5^*th*^ Generation.

Hearing Phenotype	BHL	L-CSSD	R-CSSD	NH	Total
Bilateral Normal	2	0	1	3	6
Light color (left)	2	1	1	0	4
Light color (right)	2	2	2	1	7
Bilateral pigmentation	13	7	5	0	25
Total	19	10	9	4	42

*The pig with unknown hearing phenotype was excluded from counting. The iris color of one L-CSSD and one R-CSSD individuals were not recorded and excluded from analyzing.*

### Coat Color Had No Relation With Hearing Loss

The wild type Bama Miniature pig exhibited “liang-tou-wu” coat color with black head; the hip and tail and other parts of the body exhibited white color. However, in this pedigree, we observed pigmentation. The association between hearing loss and coat color in the fifth generation is listed in Table 2. In NHs, normal and abnormal coat color accounted for 50 and 50%, respectively, while in hearing loss populations, the proportions were 7.5 and 92.5%, respectively. As shown by chi-square with Yates correction (chi-square = 2.984, df = 1), no correlation between hearing loss and coat color changes was observed (*P* = 0.0841 > 0.05). Since we also observed the pigmentation of iris color in some pigs within the pedigree, we listed the iris color change in different phenotypes (Table 3). As shown by chi-square with Yates correction (chi-square = 0.003, df = 1), *p* = 0.9568 > 0.05, hearing loss had no correlation with iris pigmentation.

**TABLE 2:** The phenotype distribution under different mating strategies in 4^*th*^ generation.

Father	Mother	BHL(%)	CSSD(%)	NH(%)	Individuals
BHL	NH (6)	1(2.86)	3(8.57)	31(88.57)	35
BHL	BHL (3)	6(37.5)	4(25)	6(37.5)	16
CSSD	CSSD (6)	19(45.24)	20(47.62)	3(7.14)	42
BHL	CSSD (2)	10(71.42)	2(14.28)	2(14.28)	14

**TABLE 3:** The Coat color and the hearing loss of 5^*th*^ Generation.

Hearing Phenotype	BHL	L-CSSD	R-CSSD	NH	Total
Normal	2	1	0	2	5
Abnormal	17	10	10	2	39
Total	19	11	10	4	44

*The individual of unknown hearing phenotype was excluded from analysis.*

### The Deaf Side of CSSDs Had No Relation With Gender

In the whole pedigree, female individuals amounted to 16 (55.17%) and male individuals 13 (44.83%). The ratio of female CSSD to male CSSD was 1.23:1, close to 1. In the whole pedigree, L-CSSDs amounted to 14 (48.28%), and R-CSSD totaled 15 (51.72%) with a ratio of 1:1.07. Among female CSSDs, the proportion of L-CSSD (9/56.25%) to R-CSSD (7/43.75%) was 1.28:1. Among the male CSSDs, the proportion of L-CSSD (5/38.46%) to R-CSSD (8/71.54%) was 1:1.6 (Figure 1D). As shown by the Fisher’s exact test, no correlation between gender and the deaf side of CSSD was observed (*P* = 0.4621 > 0.05).

### The Deaf Side of CSSDs Showed No Relation With That of Their Parents

In the fifth generation, there were 45 siblings with 21 (46.67%) CSSD individuals, 19 (42.22%) BHL individuals, 4 (9.00%) NH individuals, and 1 (2.11%) unknow phenotype (died at P1 because of diarrhea before hearing test) (Figures 1A,B). In the fifth generation, L-CSSDs were 11 (52.38%) with R-CSSD 10 (47.62%); the ratio between them was 1.1:1.

Next, we explored the relation of deaf laterality between the offspring and their parents. The paternal pig of the fifth generation was the same R-CSSD male pig; the maternal pigs included both L-CSSD and R-CSSD individuals. Under the mating strategy of Male R-CSSD with female L-CSSD, two (25.00%) of the offspring were R-CSSDs and six (75.00%) were L-CSSDs, and under the strategy of male R-CSSD mating with female R-CSSD, eight (61.64%) of the offspring were R-CSSDs and five (48.36%) were L-CSSDs (Figure 1C). As shown by the Yates corrected chi-square test, no correlation between the maternal deaf side and that of the offspring was observed (*P* = 0.2387 > 0.05).

### CSSD and BHL Presented Congenital Profound Sensorineural Hearing Loss

**FIGURE 2:**
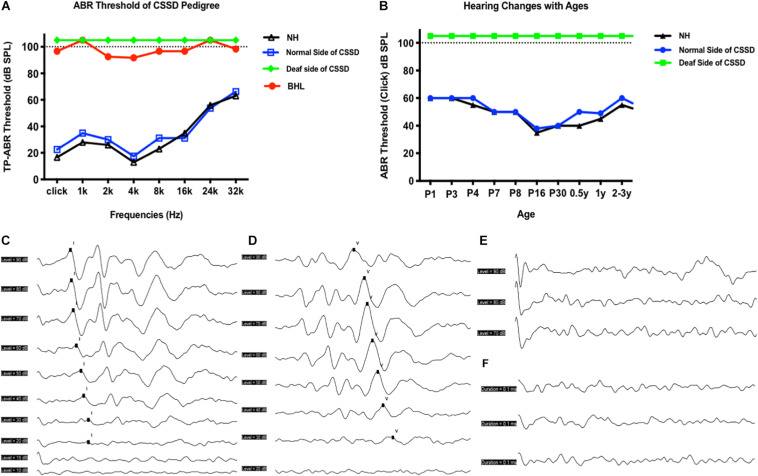
Auditory brainstem response (ABR) hearing thresholds of congenital single-sided deafness (CSSD) pedigree. **(A)** ABR thresholds from 1 to 32 kHz of different hearing phenotypes in CSSD pedigree. **(B)** ABR thresholds changes in the CSSD individuals with age compared to NHs. In Panels **(A,B)**, the threshold of 105 dB SPL means that no response was evoked at 100 dB SPL. (**C,D**) ABR waves triggered by click of NH and normal side of CSSD, respectively. (**E,F**) ABR waves triggered by click of deaf side of CSSD and bilateral hearing loss (BHL), respectively.

bilateral hearing losses and the deaf ear of CSSD individuals presented congenital profound sensorineural hearing loss since P1 through all frequencies. NHs and the normal side of CSSD showed normal hearing thresholds since P1 through all frequencies (Figure 2A). The hearing function of both normal and deaf side of CSSD remained stable through age, the normal side would not be influenced by the loss of hearing of the contralateral side (Figure 2B). In NHs and the normal side of CSSD ABR waveforms, seven waves could be evoked by each stimulus from click to 32 kHz (Figure 2C and Supplementary Figure 1). In BHLs and the deaf side of CSSD, no waveform of clear response could be identified at all frequencies (Figure 2D and Supplementary Figure 1).

**FIGURE 3:**
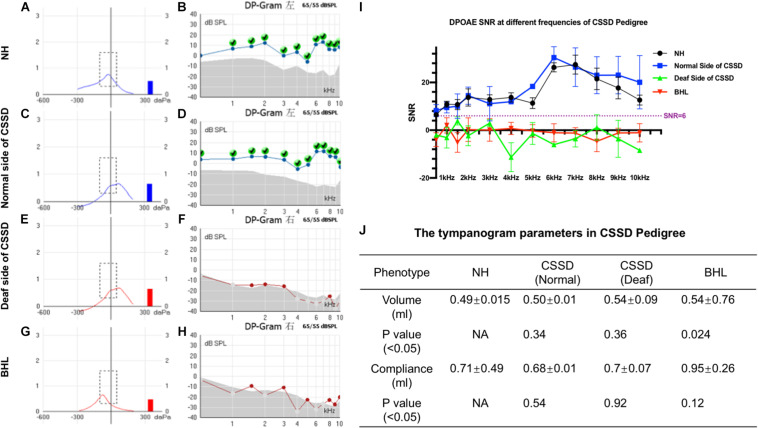
Distortion product otoacoustic emission (DPOAE) and tympanogram results of congenital single-sided deafness (CSSD) pedigree. **(A,C,E,G)** Tympanograms of each phenotype in the CSSD pedigree. **(B,D,F,H)** DPOAE results from 500 Hz to 10 kHz of each phenotype in the CSSD pedigree at P1. **(I)** Signal-to-noise ratio (SNR) of DPOAE at each frequency of different phenotypes in the pedigree. **(J)** Volume and compliance of the middle ear of each phenotype in the pedigree (*P* < 0.05 was considered statistical different).

The volume and the compliance of middle ear showed that there were no statistical differences among each group of the CSSD pedigree (Figures 3A,C,E,G,J). This result excluded the possibility of middle ear malfunction. The OHCs of NHs and normal sides of CSSDs responded well to the DPOAE stimuli from 500 to 10kHz (Figures 3B,D), while the OHCs of BHLs and the deaf sides of CSSDs showed no responses to the DPOAE stimuli (Figures 3F,H) with signal-to-noise ratio (SNR) largely below six (Figure 3I). The above result demonstrated the loss of function of OHCs since birth.

### Deaf Sides of CSSD HCs Degenerated From Apex to Base Turn and Through Age

**FIGURE 4:**
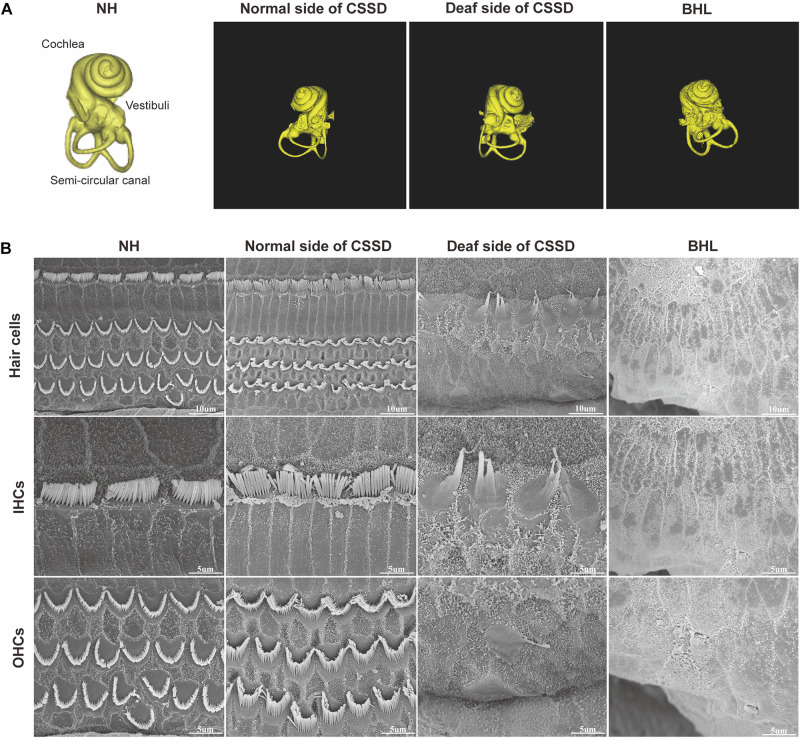
The micro-CT images of different phenotypes and SEM images of inner ear basal membrane in congenital single-sided deafness (CSSD) pedigree. **(A)** Micro-CT 3D reconstructions of the cochlear of different phenotypes in the pedigree. **(B)** SEM images of the inner ear basal membrane. The normal side of a CSSD individual exhibited normal inner ear structure as the normal hearing (NH) ones. Sporadic inner hair cells (IHCs) with fused hair bundles were left in the deaf side of CSSD individuals. In bilateral hearing loss (BHL) individuals, nearly no hair cells were left on the basal membrane. The BHL showed more severe deterioration compared to the deaf side of CSSD.

Figure 4A shows the three-dimensional (3D) reconstruction images of the cochleae in the pedigree. All cochleae exhibited normal structures: the coiling of the cochlear capsule reached three and a half turns, three semicanals were mutually vertical to each other. The cross-section showed the microstructure of the cochlear (Supplementary Figure 4). Figure 4B showed the overall pathologic changes of HCs in the pedigree. In NH and normal side of CSSD cochleae, four rows of hair cells could be observed (one row of IHCs and three rows of OHCs). The bundles of hair cells shaped like “V”. In the deaf side of CSSD, bundles were fused and HCs disorganized. For BHLs, HCs deteriorated and were replaced by non-hearing sensory cells.

**FIGURE 5:**
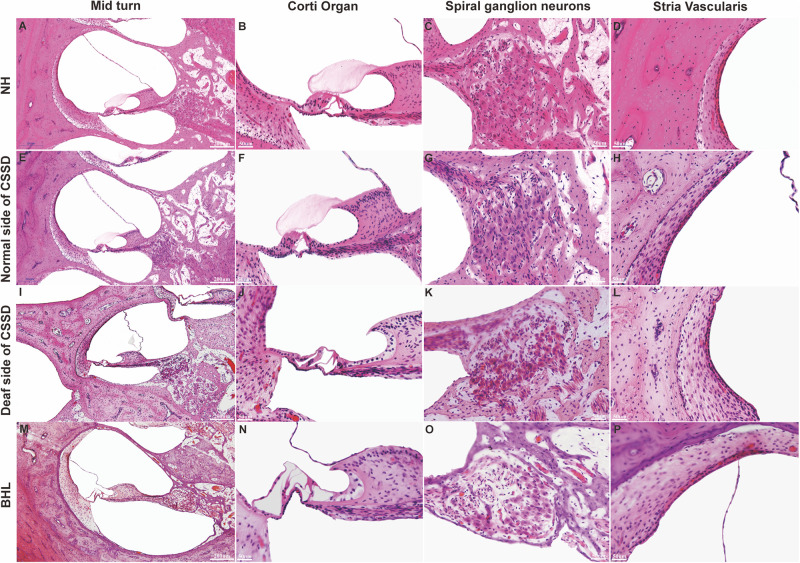
Celloidin embedding–hematoxylin–eosin (CE-HE) results of each phenotype at P8 in congenital single-sided deafness (CSSD) pedigree. Panels **(A,E,I,M)** were at low magnification (10×). The other images are all high magnification (40×). Panels **(B**,**F**,**J**,**N)** showed fine structure of the organ of Corti; panels **(J,N)** showed loss of hair cells in the deaf side of CSSD and bilateral hearing loss (BHL) individuals. Panels **(C**,**G**,**K**,**O)** showed the changes in spiral ganglion cells. Panels **(D**,**H**,**L**,**P)** showed the fine structure of stria vascularis of each phenotype in CSSD pedigree.

**FIGURE 6:**
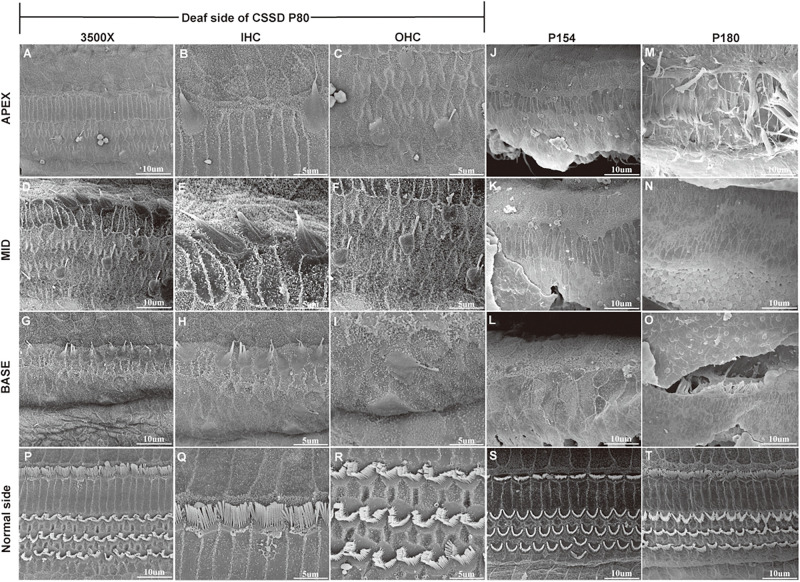
SEM results of the basal membrane of congenital single-sided deafness (CSSD) individual at different ages. **(A–I)** Images of the basal membrane of the deaf side of CSSD at P80. **(A,D,G)** Images from the apex to base turn of the cochlear at low magnification of 3,500×. **(B–I)** Inner (IHCs) and outer hair cells (OHCs) from the apex to base turn at high magnification of 8,000×. **(J–L)** Images of the basal membrane from the apex to base turn of the deaf side of the CSSD at P154; nearly no HCs were left on P154. **(M–O)** Images of the basal membrane from the apex to base turn of the deaf side of the CSSD at P180. **(P–T)** Images of the contralateral normal side of the CSSD individuals at P80, P154, and P180, respectively.

**FIGURE 7:**
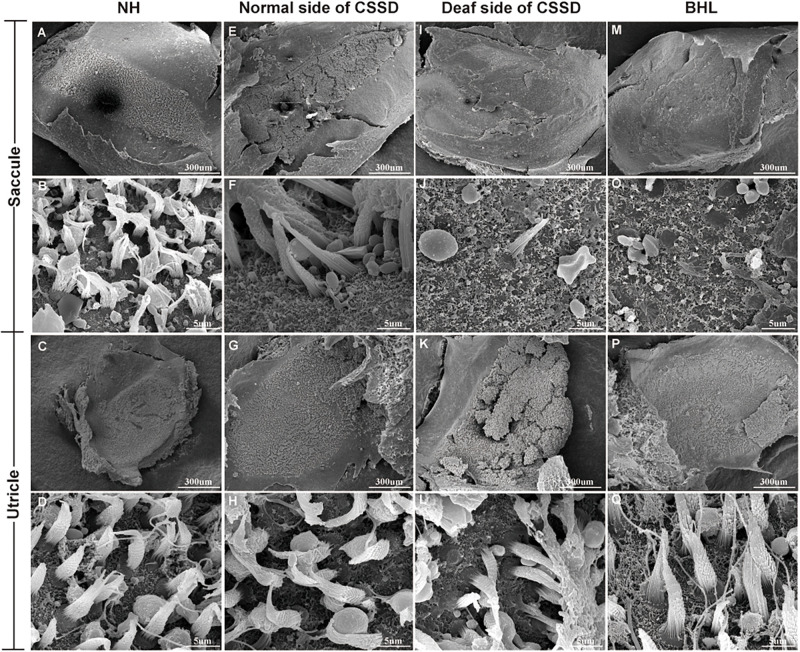
The SEM of saccule and utricle in congenital single-sided deafness (CSSD) pedigree. The deaf side of the CSSD individuals exhibited congenital hair cell malformation with sporadic hair cells left and infused hair bundles. The bilateral hearing loss (BHL) individuals showed the same pathology changes as the deaf side of the CSSD. The microstructure of the utricles in different phenotypes of CSSD pedigree were normal with normal hair cells and otolith.

In the CE-HE images of NHs and the normal sides of CSSDs, three rows of OHCs and one row of inner hair cells (IHCs) could be identified, and supporting cells like Dieter cells, Hansen cell, and inner and outer pillar cells closely and regularly arranged (Figures 5A–H). In the deaf sides of CSSDs (Figure 5I), OHCs were more vulnerable than IHCs and began to degenerate from apex to base turn through age (Figures 6A,D,G). Supporting cells loosely contacted with each other (Figure 5J) the morphology of SV and ganglion neurons remained normal in CE-HE images (Figures 5K,L). At P8, few cells were observed on the BM at apex turn, loss of OHCs could be observed on the mid turn and nearly normal BM on the base turn (Supplementary Figures 2B,F, and J). Till P80, no HCs could be observed on the BM; sporadic IHCs and OHCs with fused bundles scattered along middle and basal BM (Figures 6A–I). At P154, no HCs were left on the BM from the apex to base turn (Figures 6J–L). At P180, fibers replaced the BM cells at the apex turn; vestibular membrane was closely contacted with BM and only pillar cells were left under the BM with supporting cells being replaced (Figures 6M–O). The morphology of hair cells of each turn in good hearing ear of CSSDs remained normal at P80, P154 and P180 (Figures 6P–T). This part demonstrated that the BM of the deaf sides of CSSDs degenerated from apex to base turn through age, and the pathology began before birth which coincided with the hearing phenotype. Meanwhile, the morphology of saccule and utricle of NH and normal side of CSSD were normal with identifiable hair cells and otolith (Figures 7A–H). The saccule of the deaf side of CSSDs was identical to NH while the utricle showed degenerated hair cells (Figures 7I–L).

### BHL Showed the Same, but More Severe and Rapid Pathological Progress as CSSD

In the BHLs, HC damage progressed the same trajectory as the CSSD but was more severe and rapid compared to the deaf sides of CSSDs. At P1, hair bundles of IHCs at the apex turn were fused; OHCs were disorganized with fused bundles (Supplementary Figures 4A–C). IHCs of the middle and basal turn exhibited nearly normal structure. OHCs at the middle were disorganized and began to degenerate (Supplementary Figures 4D–F). Till the basal turn, IHCs were in normal structure and OHCs were in three lines, but bundles in some OHCs began to fuse (Supplementary Figures 4G–I). At P8, hair cells had degenerated, only the nucleus of inner hair cell could be seen and the number of supporting cells decreased which led to the disruption of the structure of organ of Corti (Figures 5M–P). At P14, no HCs could be observed at the apex turn; few HCs could be identified at the middle turn, and nearly all HCs could be seen at the basal turn; bundles of remained HCs were all fused (Supplementary Figures 4J–L). Till P30, no HCs were left on the basal membrane (Supplementary Figures 4M–O). The SEM results coincided with the CE-HE results (Supplementary Figure 3). The above results demonstrated that BHL follow the same pathological trajectory as CSSD, but the progress was more rapid and severe. As for the vestibular organ, the structure of saccule remained normal and the hair cells in utricle had degenerated since P1 (Figures 7M,O–Q).

### The Ganglion Cells of NS of CSSD Declined Compared to the NH

**FIGURE 8:**
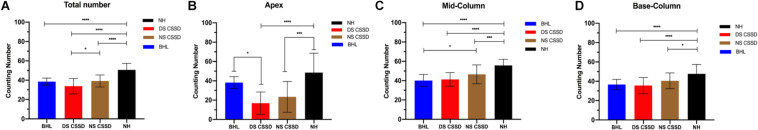
Spiral ganglion cell counting at P30. **(A)** Averaged total ganglion cell counting number from the apex to base turns. **(B)** Ganglion cell counting number in the apex turn in different hearing phenotypes. **(C)** Ganglion cell counting number in the mid turn in different hearing phenotypes. **(D)** Ganglion cell counting number in the base turn in different hearing phenotypes. *p < 0.05, **p < 0.01, ***p < 0.001, ****p < 0.0001.

The ganglion cells counting number of BHL, deaf side (DS) of CSSD, and normal side (NS) of CSSD showed significant decrease compared to the NH and that of the DS of CSSD showed significant reduction compared to the NS of CSSD; about 76, 77.4, and 66.7% cells survived for BHL, DS of CSSD, and NS of CSSD, respectively (Figure 8A and Table 4). For the apex turn, both DS and NS of CSSD showed sever cell loss and significantly less than the BHL individuals (Figure 8B). For the mid and base turn, ganglion cells of BHL and DS of CSSD both showed clear reduction compared to the NS of CSSD and NH individuals (Figures 8C,D). The loss of ganglion cells was most severe in the apex turn for the CSSD individuals and was equal for each turn for BHL individuals.

**TABLE 4:** The ganglion cell counting number of each hearing phenotype at P30.

Hearing Phenotype	BHL	NS-CSSD	DS-CSSD	NH
Apex	38.2 ± 6.14	23.40 ± 15.99	16.83 ± 11.56	48.53 ± 19.99
Mid	40.18 ± 6.31	46.58 ± 9.76	41.31 ± 7.09	55.71 ± 6.43
Base	36.59 ± 6.35	40.46 ± 8.25	35.54 ± 8.38	47.67 ± 9.60
Total	38.50 ± 3.71	39.21 ± 6.27	33.76 ± 8.06	50.65 ± 6.75

### The Stria Vascularis Degenerated Over Time After Birth

**FIGURE 9:**
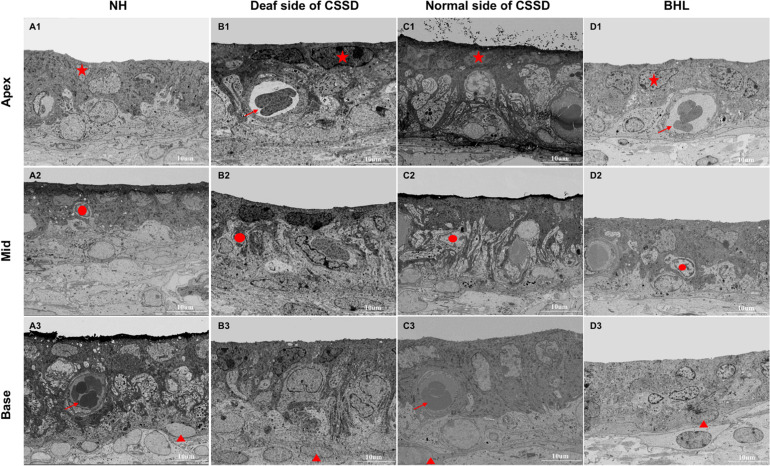
The SEM images of stria vascularis of different phenotypes in the congenital single-sided deafness (CSSD) pedigree at P30. **(A1-D3)** SEM images of the stria vascularis at different turns of normal hearing (NH), deaf side (DS) of CSSD, normal side (NS) of CSSD, and bilateral hearing loss (BHL). For NH, three layers of cell could be clearly identified: star, marginal cell; circle, intermediate cell; and triangle, basal cell. Vascularis (arrow) could be seen in some parts of the stria vascularis. No obvious malformation was observed in the DS of CSSD and NS of CSSD; in the BHL, the three layers of cells were disorganized, and the stria vascularis was thinner than normal.

In NHs, the stria vascularis was composed of three layers of cells: marginal cells, intermediate cells, and basal cells. Marginal cells (star) were tightly attached with each other by gap junction. Basal cells (triangle) were interconnected to separate stria vascularis (SV) from spiral ligament. Intermediate cells (circle) were in the middle layer in which melanin spots could be identified. At P30, the SV of NHs and both sides of CSSDs were of normal structure, while in BHL, SV was disorganized with only two layers of cells survived (Figure 9).

The structure of SV degenerated through age. In DS of CSSD cochleae, the structure of SV remained normal till P154. At P180, the SV was dominated by marginal and basal cells; the intermediate cells declined (Supplementary Figure 6). In BHL cochleae, the structure of the SV remained normal. However, at P30, the cells in SV were disorganized, the same as that in DS of CSSD. The SV was composed of two layers of cells, mainly marginal and basal cells; intermediate cells were hardly identified (Supplementary Figure 7).

## Discussion

Sensorineural hearing loss is often induced by loss of HCs and SGNs in the inner ear cochlea (Liu et al., 2016, 2019; Zhong et al., 2020). HC transduces the sound waves into electric signals (Qi et al., 2019), while SGNs transfer these signals into the auditory cortex to have the hearing ability (Guo et al., 2016, 2019, 2020). WHO reported that 466 million people are suffering with hearing loss worldwide, caused by genetic factors, infectious diseases, chronic cochlear infections, aging, exposure to noise, and ototoxic drugs (He et al., 2017, 2020; Tan et al., 2019; Zhang et al., 2020; Zhou et al., 2020; Lv et al., 2021); for children from 6 to 19 years old, the estimated CSSD incidence was 0.7–0.8%. In this study, we reported a Bama miniature pedigree with high and stable incidence of CSSD, which reached 46.67% under the mating mode of paternal CSSD with maternal CSSD pigs. In this pedigree, the hearing phenotypes varies with different mating modes. Under the mode of CSSD with CSSD, BHLs accounted for 42.22% and CSSDs 46.67%, NHs only accounted for 9%. All hearing loss individuals exhibited congenital severe sensorineural hearing loss with no ABR, DPOAE response being evoked since birth. Normal tympanogram had excluded the possibility of middle ear diseases. Phenotype of hearing loss was not correlated with coat color and gender. The deaf side of the offspring had no relation with the deaf side of their parents. This porcine model highly mimicked the human non-syndromic CSSD in the clinics and might be the first porcine model with high and stable presence rate of CSSD being reported in the world.

In this paper, we studied the auditory physiology and pathology of BHL, NH, and CSSD individuals. All the deaf cochlear showed cochlear–saccular degeneration, also known as Scheibe dysplasia. For DS of CSSD and BHL, bundles of hair cells were fused since birth, and hair cells degenerated from the apex to the base. In the CSSDs, sporadic hair cells could be identified at P80–P154; basal membrane was replaced by the epithelial cells. BHL showed the same but more severe and rapid pathological process; till P30, hair cells and supporting cells could not be identified, and the Reissner’s membrane tensely attached to the basal membrane. Only sporadic pillar cells survived. The ipsilateral saccule exhibited degenerated hair cells since P1. Scheibe dysplasia had been proven to occur in many animals, like deaf white cats, Dalmatian dogs, waltzing guinea pigs, and mice (Schuknecht et al., 2009). It was reported that the Scheibe dysplasia occurred in 70% of the cases with hereditary hearing loss (Lalwani et al., 1997), which is similar to our porcine deafness model. The structure of SV in the deaf ear degenerated through age; in CSSDs, the disorganization could be observed from P180, while in BHL, the malformation presented no later than P30. This time course corresponds to that of the hair cells, which might indicate that the SV degeneration was not responsible for hearing loss but an outcome of hearing loss.

For the degeneration of the ganglion, we did not have enough animals at different ages, so we chose pigs of P30 to analyze the pathology of ganglion cells. The pathology changes in each hearing phenotype were uniform in this pedigree. Significant reduction in ganglion cells of BHL, DS of CSSD, and NS of CSSD could be observed. For BHL, the ganglion cell counting number at each turn decreased proportionately. For CSSDs, the most severe cell loss occurred in the apex turn; we should also pay attention to the NS of CSSD; the total ganglion cell number and that of each turn showed a significant reduction compared to NHs. This might indicate that the NS of CSSD might be vulnerable to factors that harm hearing like noise exposure and ototoxic drugs. This coincided with the reports of the vulnerability of the good hearing ear of CSSD humans.

We also calculated the ganglion cell number of BHL at P1; the mean value for apex, mid, and base turns were 38.20 ± 6.14 (*P* = 0.19 > 0.05), 40.77 ± 7.70 (*P* < 0.0001), 36.00 ± 6.06 (*P* < 0.0001), respectively, and the counting number at apex turn showed no significant differences with the NH. Meanwhile, the organ of Corti was flattened, and supporting cells vanished at the apex and mid turn at P1 (Supplementary Figure 3). For the deaf side of the CSSD individuals, the counting number of ganglion cells for the apex, mid, and base turns were 11.7 ± 8.47 (*P* < 0.001), 37.10 ± 7.16 (*P* < 0.001), and 31.30 ± 7.14 (*P* < 0.001), while the organ of Corti showed the same but much milder pathological changes. These results indicated that the pathological process started from embryogenesis period because the degeneration of supporting cells occurring after the acquired hearing loss would trigger the neural degeneration at least 2 weeks away (Schuknecht et al., 2009).

Congenital single deafness of white cat, dog, and horse were also reported. In canine deafness model, the unilateral deafness reached 21.9% in a Dalmatian breed; however, no underlying inheritance mechanism and contributing genes had been revealed. Hayward et al. (2020) did genome-wide association study (GWAS) in 3 canine breeds; 14 suggestive genes were found, but none was located in the areas causing WS as their symptoms indicated, and the genes had no overlap between species. Numerous labs had tried to find the causative gene for the natural deafness canine breeds; only few identified genes, like SOX10 (Hansen et al., 2010) and OTOF (Cargill, 2004), were verified genes responsible for human deafness. One possibility of why no suggestive genes overlap among breeds was that they were caused by different underlying genetic mechanisms. Therefore, GWAS could not be done by using samples from different pedigree; the best would be samples from one inbred pedigree.

In our study, we also could not locate the possible responsible genes and identify the possible inheritance mode. Neither a dominant nor a recessive simple Mendelian mode of transmission could be proven by chi-square analysis in the pedigree. Gender differences and correlation between coat color and hearing loss were also not observed; the deaf side of CSSD had no relation with their parent deaf side. Unlike the DWC, deaf dog, and horse breeds with pigmentation and iris color change, the pigs in our pedigree presented non-syndromic hearing loss and cochlear–saccular degeneration, much more like the CSSD patients in the clinics. However, the interaction between genes associated with coat and iris color and that with hearing loss was unclear. The genetics underlying the disease is very complex. One reason is that the matching rate of human and animal deafness causative genes is very low; the other is that other elements also affect the expression, like epigenetic and transcriptomic factors.

Pigs have become an important biomedical model due to their genetic, anatomical, and physiological similarities with human, as well as the short generation interval (∼114 days), broad availability, large litter size, and lower ethical concern because they are of a kind of food source instead of companion animals like cats and dogs (Schook et al., 2015). Pigs are also widely used in the studies of human disease, like cancer model, cardiovascular model, metabolic and gastrointestinal disease model, and hearing loss model. The porcine cochlear anatomy and auditory physiology highly resembles that of human. Unlike rodent lab animals, the porcine cochlear size was much larger and may provide us a chance to study the hearing and behavioral changes and the cortex reorganization brought by CI in CSSD animals, which might give an indication on the time window of CI in CSSD children clinically. Additionally, the pig’s brain resembles that of the human in size, anatomy, development, and importantly the cognitive development (Lind et al., 2007; Elmore et al., 2012). This would allow us to gain a deep insight into the impact of CSSD on cognitive development and the benefits of CI. In the future study, pedigree genome and RNA sequencing will be combined to find the possible responsible genes that might reveal the mechanism underlying CSSD.

## Data Availability Statement

All the relevant data is contained in this article. The original contributions presented in the study are included in the article/supplementary material, further inquiries can be directed to the corresponding authors.

## Ethics Statement

The animal study was reviewed and approved by The Ethics Committee of Chinese PLA General Hospital. Written informed consent was obtained from the owners for the participation of their animals in this study.

## Author Contributions

WR, CX, and F-JZ mainly conducted the experiment. YW, T-TL, C-HL, X-YZ and L-LW were mainly in charge of breeding and maintaining the pedigree. YZ mainly conducted the SEM and TEM studies and illustrated the relative results. HZ, W-WG, PJ, and S-MY designed the study, analyzed the data, and illustrated the pathology results of the pedigree. WR finally contributed to the writing of the article, figures, and tables. All authors contributed to the article and approved the submitted version.

## Conflict of Interest

The authors declare that the research was conducted in the absence of any commercial or financial relationships that could be construed as a potential conflict of interest.

## Supplementary Material

Supplementary Figure 1 The ABR waveform at different frequencies of the CSSD individual. Panels **(A–H)** showed that on the deaf side of CSSD, no ABR response was triggered from 1kHz to 32kHz. Panels **(I–P)** showed that on the normal side of CSSD, identifiable ABR responses were evoked and the thresholds of each frequency showed no statistical difference from the NHs.

Supplementary Figure 2 The CH-HE images of CSSD cochlear at P8. Panels **(A–D)** showed the images of the apex turn, no sensory cells were left on the BM. Panels **(E–H)** showed the images of the mid turn, supporting cells and OHCs were left on the BM. Panels **(I–L)** showed the images of the base turn, IHC, OHC and supporting cells all could be seen. Panels **(M–P)** showed the contralateral normal side of CSSD individual.

Supplementary Figure 3 The CH-HE images of BHL cochlear at P30. Panels **(A–D)** showed the images of the apex turn, no sensory cells were left on the BM. Panels **(E–H)** showed the images of the mid turn, only supporting cells were left on the BM. Panels **(I–L)** showed the images of the base turn, sporadic hair cells could be seen at P30.

Supplementary Figure 4 CH-HE image of 4X magnification. This specimen was the normal side of CSSD with mid-modiolus, the coiling of the cochlear adequate reached three turns. In the above image, 1 and 2 were apex turn, 3 and 4 were mid turn, 5 and 6 were base turn and 7 was the very end of the basal membrane named hook region. Ganglion cells inside the black dotted line were counted.

Supplementary Figure 5 SEM results of Basal membrane of BHL individual at different ages. Panels **(A,D,G)** were images of P1 BHL cochlear from apex to base turn at low magnification of 3500X. Panels **(B–I)** showed the inner and outer hair cells at higher magnification of 8000X. Panels **(J–L)** were images of P14 BHL cochlear from apex to base turn. Panels **(M–O)** showed images of P30 BHL cochlear from apex to base turn.

Supplementary Figure 6 TEM results of Stria Vascularis of the deaf side of CSSD individuals at different ages. Panels **(A1-3,B1-3,C1-3)** were stria vascularis images of DS of CSSD from apex to base turn at magnification of 3500X at P80, P154, and P180. The structure of stria vascularis at P80 was nearly normal, three layers of cells (marginal, intermediate and basal cells) were identified. Till P180, the stria vascularis remained nearly normal.

Supplementary Figure 7 TEM results of Stria Vascularis of BHL individual at different ages. Panels **(A1–3,B1–3,C1–3)** were stria vascularis images of P1 BHL cochlear from apex to base turn at magnification of 3500X at P1, P14, and P30. The structure of stria vascularis at P1 was normal, three layers of cells (marginal, intermediate and basal cells) were identified. Till P30, the stria vascularis was disorganized, only the marginal cells could be clearly identified.
