# Supplementary figures and images for: A Porcine Congenital Single-Sided Deafness Model, Its Population Statistics and Degenerative Changes
Source: Front Cell Dev Biol. 2021 Jun 11;9:672216. doi: 10.3389/fcell.2021.672216 (PMC8226144; doi:10.3389/fcell.2021.672216)

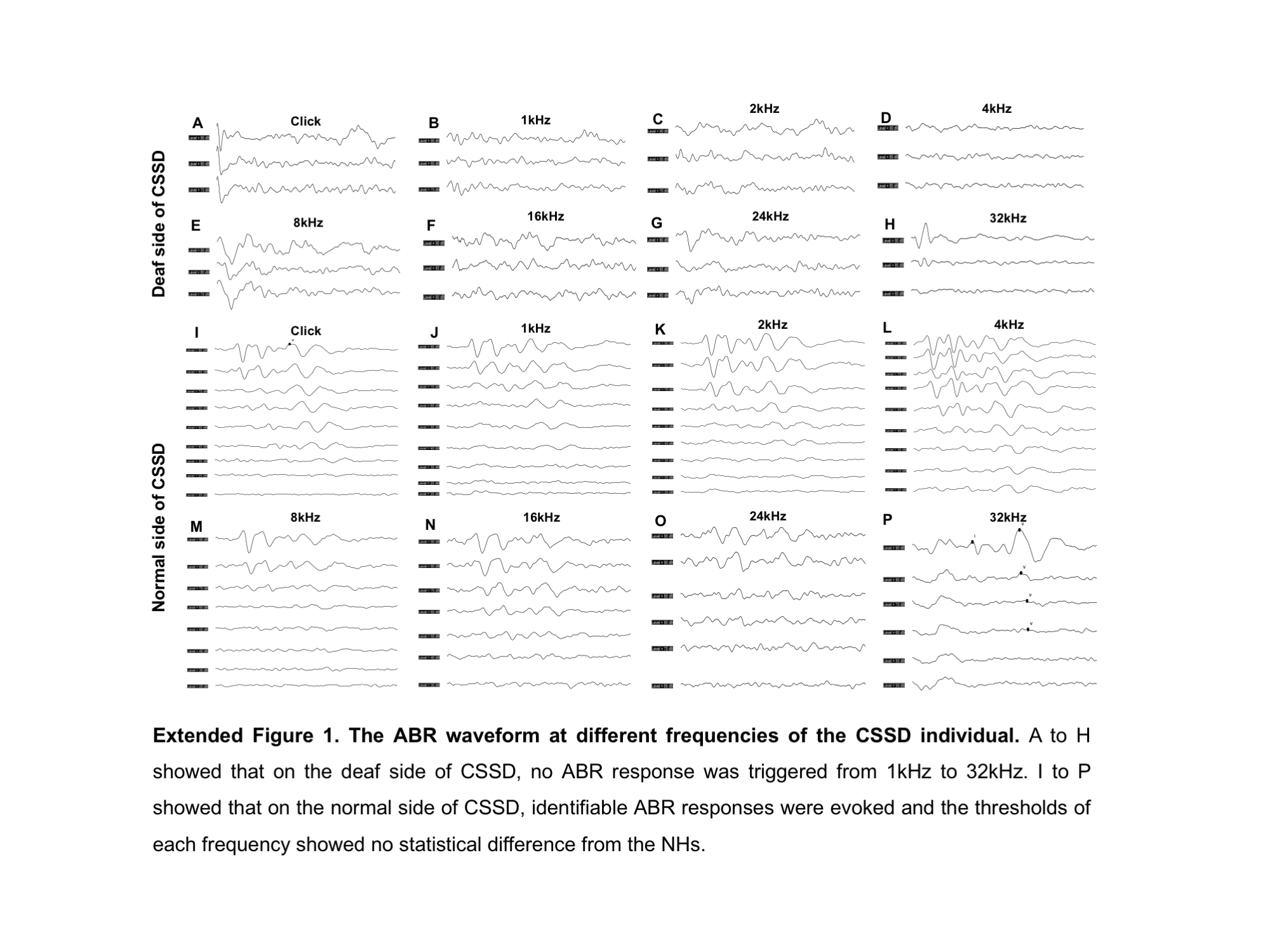

Supplement: Supplementary Figure 1 — The ABR waveform at different frequencies of the CSSD individual. Panels (A–H) showed that on the deaf side of CSSD, no ABR response was triggered from 1kHz to 32kHz. Panels (I–P) showed that on the normal side of CSSD, identifiable ABR responses were evoked and the thresholds of each frequency showed no statistical difference from the NHs. [file Image_1.TIFF]

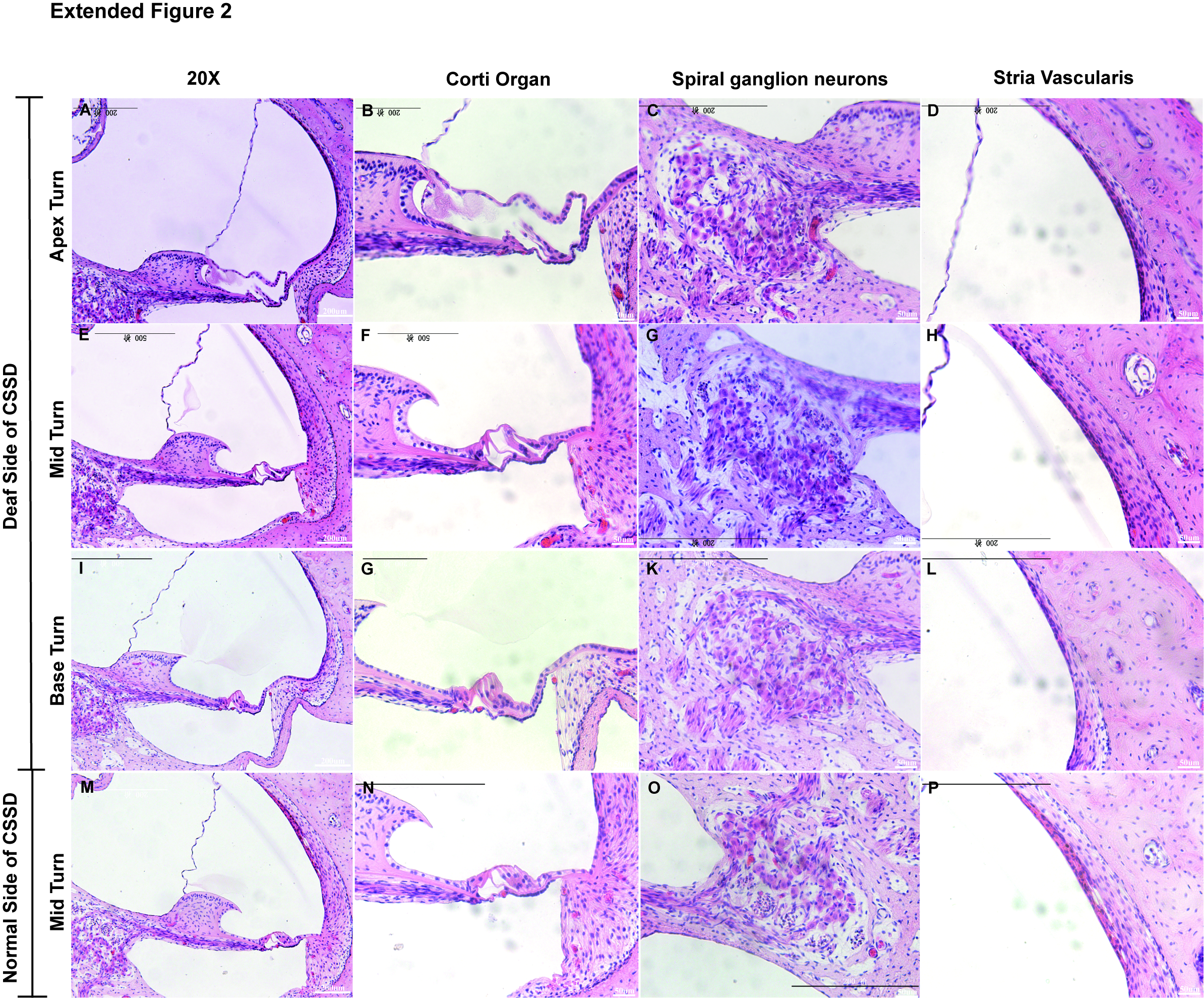

Supplement: Supplementary Figure 2 — The CH-HE images of CSSD cochlear at P8. Panels (A–D) showed the images of the apex turn, no sensory cells were left on the BM. Panels (E–H) showed the images of the mid turn, supporting cells and OHCs were left on the BM. Panels (I–L) showed the images of the base turn, IHC, OHC and supporting cells all could be seen. Panels (M–P) showed the contralateral normal side of CSSD individual. [file Image_2.TIF]

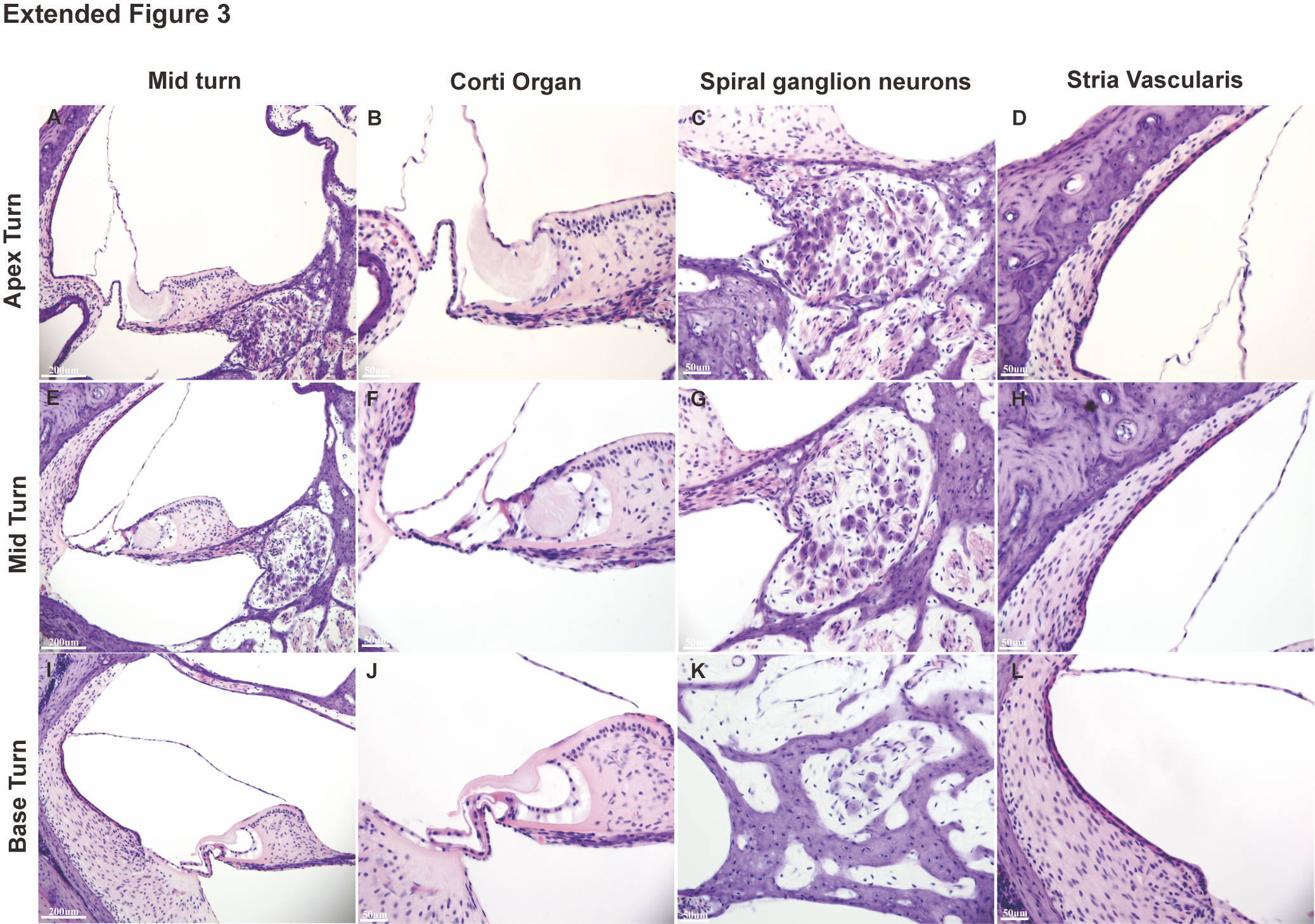

Supplement: Supplementary Figure 3 — The CH-HE images of BHL cochlear at P30. Panels (A–D) showed the images of the apex turn, no sensory cells were left on the BM. Panels (E–H) showed the images of the mid turn, only supporting cells were left on the BM. Panels (I–L) showed the images of the base turn, sporadic hair cells could be seen at P30. [file Image_3.TIFF]

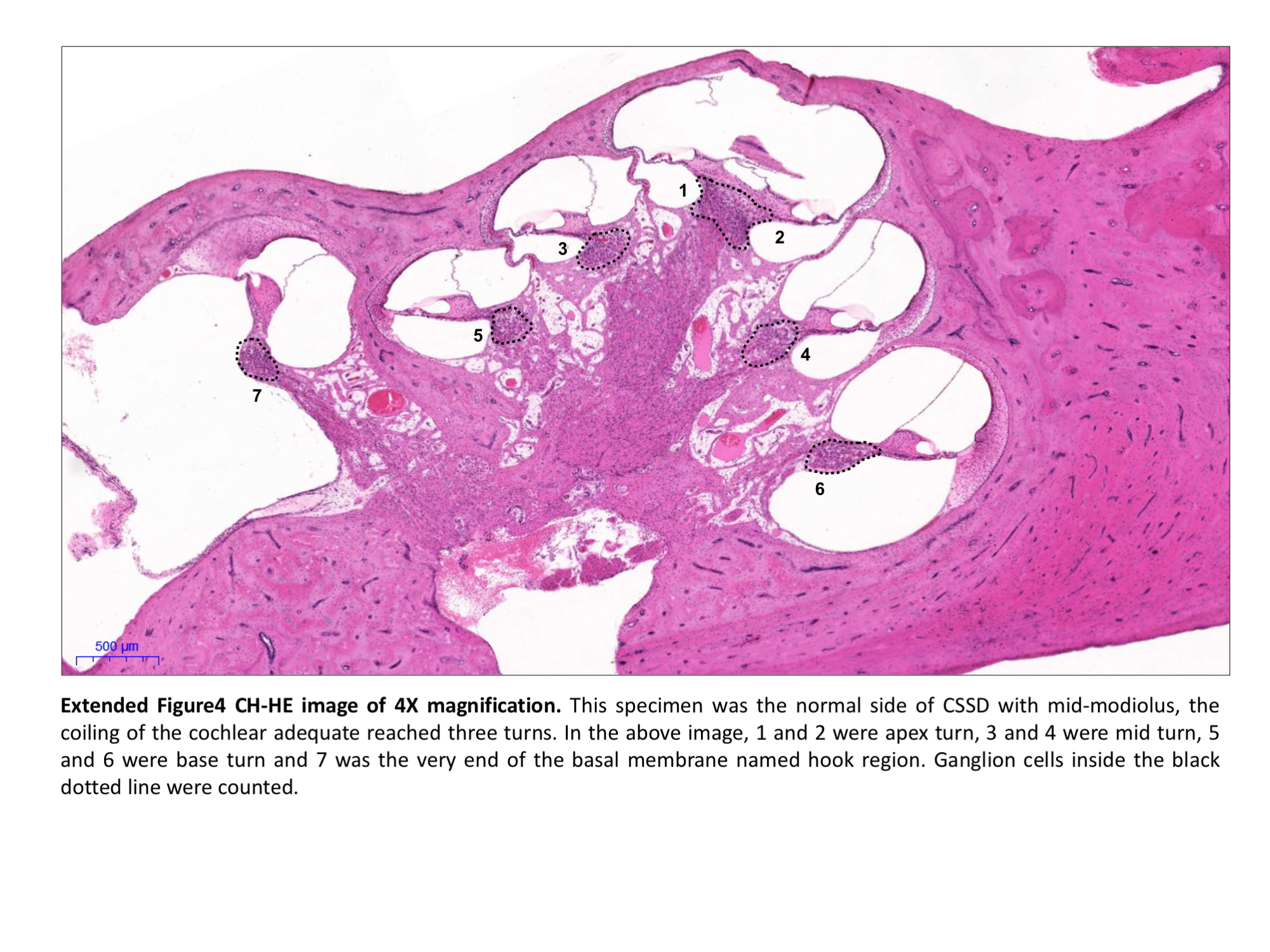

Supplement: Supplementary Figure 4 — CH-HE image of 4X magnification. This specimen was the normal side of CSSD with mid-modiolus, the coiling of the cochlear adequate reached three turns. In the above image, 1 and 2 were apex turn, 3 and 4 were mid turn, 5 and 6 were base turn and 7 was the very end of the basal membrane named hook region. Ganglion cells inside the black dotted line were counted. [file Image_4.TIFF]

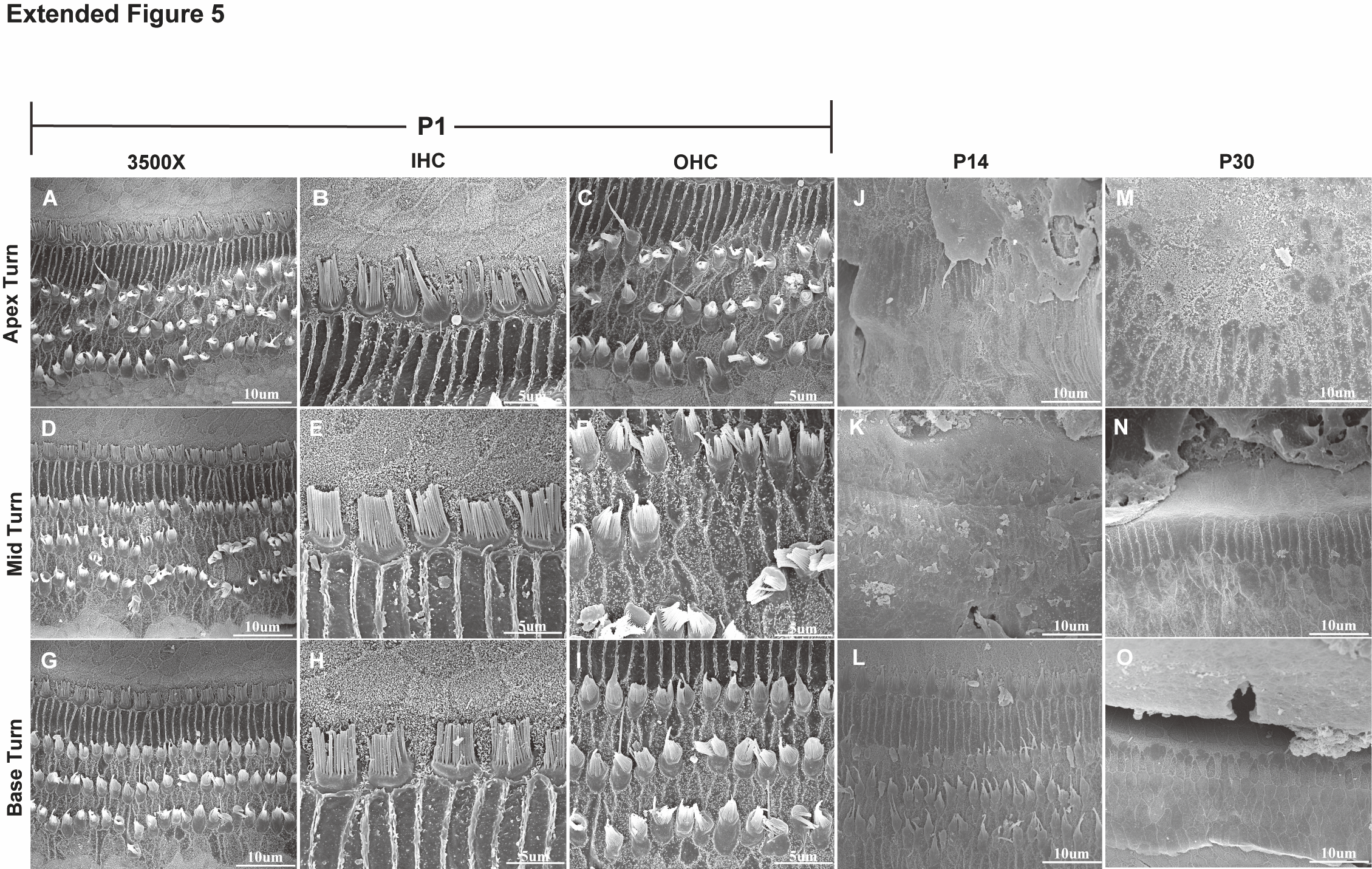

Supplement: Supplementary Figure 5 — SEM results of Basal membrane of BHL individual at different ages. Panels (A,D,G) were images of P1 BHL cochlear from apex to base turn at low magnification of 3500X. Panels (B–I) showed the inner and outer hair cells at higher magnification of 8000X. Panels (J–L) were images of P14 BHL cochlear from apex to base turn. Panels (M–O) showed images of P30 BHL cochlear from apex to base turn. [file Image_5.TIFF]

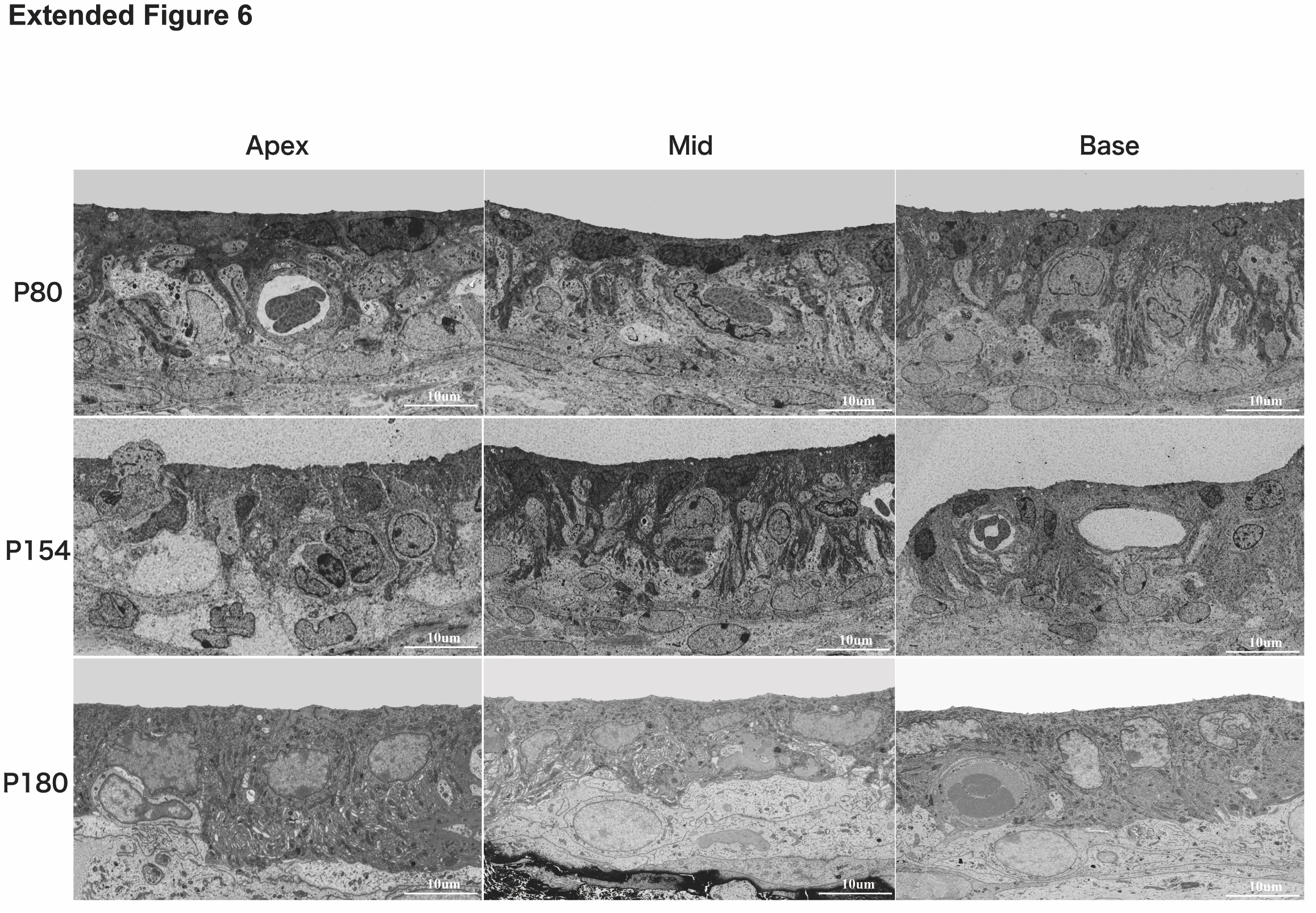

Supplement: Supplementary Figure 6 — TEM results of Stria Vascularis of the deaf side of CSSD individuals at different ages. Panels (A1-3,B1-3,C1-3) were stria vascularis images of DS of CSSD from apex to base turn at magnification of 3500X at P80, P154, and P180. The structure of stria vascularis at P80 was nearly normal, three layers of cells (marginal, intermediate and basal cells) were identified. Till P180, the stria vascularis remained nearly normal. [file Image_6.TIFF]

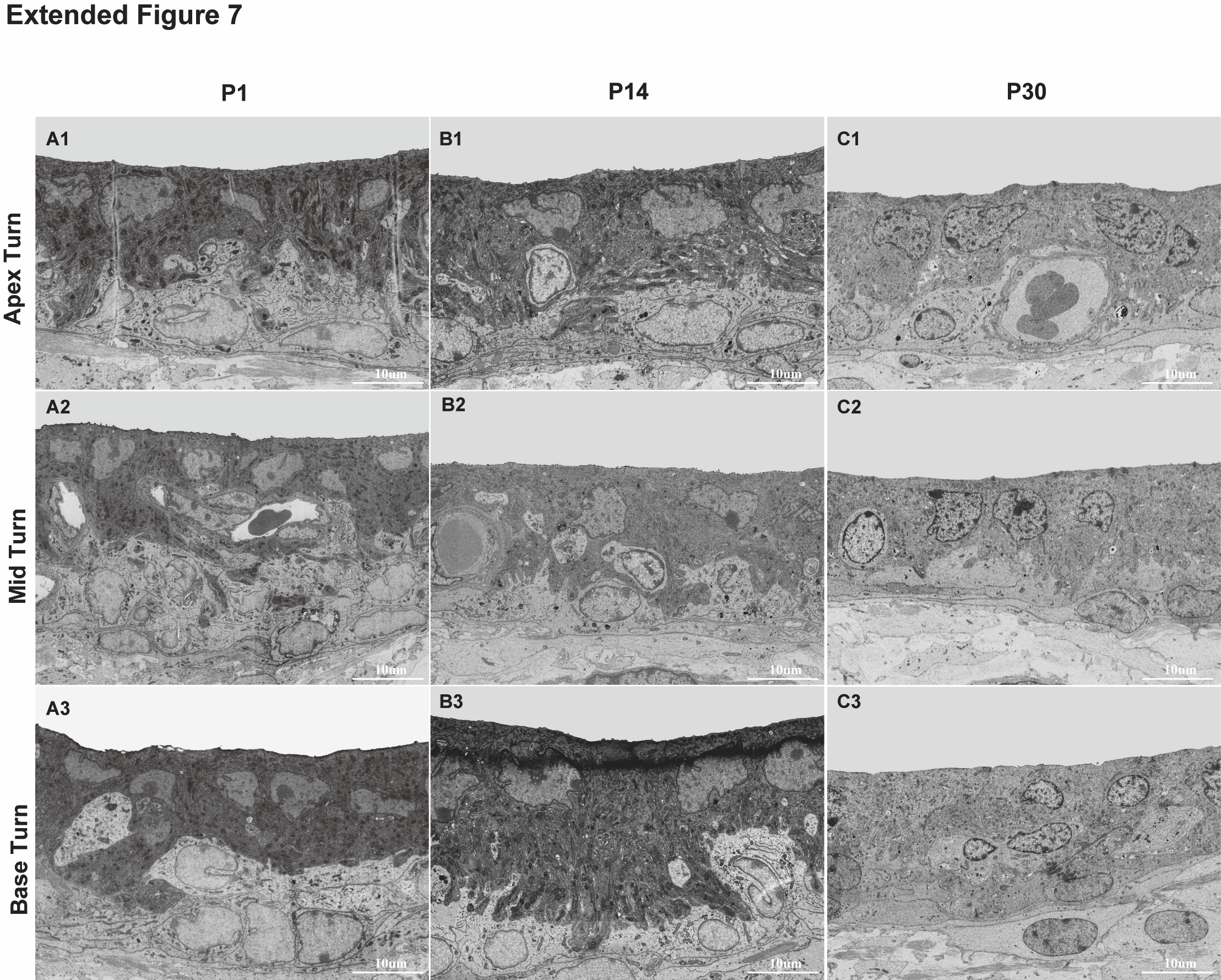

Supplement: Supplementary Figure 7 — TEM results of Stria Vascularis of BHL individual at different ages. Panels (A1–3,B1–3,C1–3) were stria vascularis images of P1 BHL cochlear from apex to base turn at magnification of 3500X at P1, P14, and P30. The structure of stria vascularis at P1 was normal, three layers of cells (marginal, intermediate and basal cells) were identified. Till P30, the stria vascularis was disorganized, only the marginal cells could be clearly identified. [file Image_7.TIFF]
